# A hybrid CNN-ViT based framework for automatic traffic actions detection in smart cities

**DOI:** 10.1371/journal.pone.0339796

**Published:** 2026-01-16

**Authors:** Mucahit Karaduman, Neunggyu Han, Gulsah Karaduman, Muhammed Yildirim, Yongwon Cho, Yunyoung Nam

**Affiliations:** 1 Department of Software Engineering, Malatya Turgut Ozal University, Malatya, Turkey; 2 Department of ICT Convergence, Soonchunhyang University, Asan, Republic of Korea; 3 Department of Computer Engineering, Firat University, Elazig, Turkey; 4 Department of Computer Engineering, Malatya Turgut Ozal University, Malatya, Turkey; 5 Department of Computer Science and Engineering, Soonchunhyang University, Asan, Republic of Korea; 6 Department of ICT Convergence, Soonchunhyang University, Asan, Republic of Korea; Instituto Politecnico Nacional, MEXICO

## Abstract

It is crucial to automatically detect traffic accidents and hazardous situations in a timely and accurate manner. In this way, both individual security will be ensured and significant contributions will be made to economic efficiency and sustainable urban life. Millions of people die in traffic accidents every year. This situation also places an additional burden on health systems and will lead to many undesirable consequences. Early detection of events such as traffic density, accidents, and road closures accelerates emergency response processes, regulates traffic flow, and prevents secondary accidents. Therefore, artificial intelligence-supported automatic systems stand out as a key component of smart cities. This study aims to detect traffic accidents and traffic situations automatically. For this purpose, feature extraction was performed with five Convolutional Neural Network (CNN) and five Vision Transformer (ViT) based models. Then, the features obtained from these models were evaluated in different classifiers. The ViT model and the CNN model, which yielded the most successful results, served as the base for the proposed model. The features obtained from the best ViT model and CNN model were combined to bring together different features of the same image. Then, these features were classified into eight different categories using various classifiers. It was observed that the proposed model produced more successful results than the ten models whose preliminary results were obtained in the study. The accuracy value of the proposed model was 96.88%. This value is promising for future studies and plays a strategic role in terms of sustainability and enhancing the quality of life in smart cities.

## 1. Introduction

Due to urbanization, the increase in motor vehicles, and the accompanying higher traffic density, traffic problems—which have become an integral aspect of human life—are worsening. Furthermore, these issues are made worse by the deployment of autonomous vehicles and smart cities. Accidents are the most significant of these issues; among their effects on people are the time, life, and property losses they inflict, the pollution they contribute to the environment, and the financial losses they cause. These factors have made it vital to improve the safety of the transportation system, particularly in smart cities. To maintain traffic flow and establish a secure environment, artificial intelligence and sensor systems are being increasingly incorporated into our daily lives. Since smart transportation systems in smart cities carry security and privacy risks in the transmission and recording of data, studies are being carried out to eliminate these problems [1–4].

Conditions, including vehicle detection, collision detection, and road conditions, are evaluated during traffic studies. The goal of studies that typically involve processing time series, sensor data, and photographs is to provide solutions for intelligent transportation. The use of Convolutional Neural Network (CNN) and Transformer designs has become commonplace in this discipline due to the success of deep learning architectures in recent years. Using information from social media, Ali et al. have developed a system for detecting and analyzing traffic accidents in real-time. Bidirectional Long Short-Term Memory (Bi-LSTM) and Ontology and Latent Dirichlet Allocation (OLDA) were utilized to extract meaningful information from raw social media data, enabling the classification of texts with 97% accuracy [5]. The Surveillance-Oriented Traffic Accident Detection (So-TAD) dataset was developed by Chen et al. The collection comprises 282 traffic collision occurrences, 2,186 samples, and photographs, along with temporal and location information. The Three-Channel Scene Feature Fusion GAN (TCFF-GAN) architecture they created utilized the scene’s information and change characteristics, yielding an AUC value of 71.25% based on cosine similarity [6]. Moriano et al. proposed a model to detect traffic accidents quickly and reliably. In the model, they suggested that fast detection could be achieved with traffic data received from neighboring sensors. To this end, they attempted to implement detection using machine learning methods, incorporating traffic information from radar sensors, as well as light and weather data. With the XGBosst algorithm, they managed to make correct detection with an 83% AUc rate 1 minute after the accident. This study demonstrated that the accident detection rate increases with the use of neighboring sensors [7]. Zavantis et al. researched the necessity of automatic accident detection systems. They demonstrated that this system can reduce the time loss experienced in reaching the scene and managing the problem, thereby increasing the chance of survival for accident victims. Injuries can be prevented from reaching serious dimensions with prompt emergency intervention. In addition, since the system will collect preliminary information such as the number of passengers, vehicle type, and health status related to the accident, it has been concluded that the necessary intervention planning can be made more appropriately [8]. Kumar et al. proposed a four-stage method for the automatic detection of accidents. While these stages are proposed as data preparation, model training, feature extraction, and road segmentation, Average Annual Daily Traffic (AADT) is used for segmentation. It is observed that the highest performance is achieved with Histogram of Oriented Gradients (HOG) for feature extraction. Model training accuracy is increased with Deep Metric Learning (DML), and a result of 95.40% accuracy has been obtained [9]. Nusari et al. utilized YOLOv9 and YOLO-NAS for real-time object detection in their accident detection system, where the YOLO-NAS-L model achieved notable results with 85% mAP 0.50 and 70.1% mAP 0.50:0.95. In comparison, the YOLOv9-C model provided higher accuracy with 92.7% mAP 0.50 and 86% mAP 0.50:0.95 values [10]. Karim et al. used a hybrid approach based on YOLOv8 and Deep-SORT to detect traffic incidents in smart transportation systems. By training the YOLOv8 model with a dataset and performing real-time monitoring with Deep-SORT, they detected traffic incidents with 98.4% accuracy [11]. Albekairi et al. proposed a Displacement Region Recognition Method and achieved 95.68% accuracy in license plate recognition in foggy images [12]. Yu et al. proposed a multi-faceted analysis that includes details such as the type of accident, time-space information, and severity level, as well as the accident occurrence, by defining a fine-grained accident detection task. A 3D Video Transformer-based architecture that integrates RGB and optical flow data was created for this purpose. For this study, a fine-grained Accident Detection (FAD) dataset was created. Experimental findings demonstrated that the model is more successful for in-depth analysis than traditional binary classification techniques and can extract significant information from the video [13]. Using a low-power, long-range LoRa-based architecture with GPS-enabled LoRa modules installed in cars and ultrasonic collision sensors, Vinodhini et al. present a model for more efficient emergency services in the event of an accident. By sending incident data to the cloud, the model directs users to the closest hospital. LoRa technology is more effective than other LPWAN networks in terms of low latency, broad coverage, low energy consumption, and low cost. With their study, they have made significant contributions to the development of IoT-based accident response systems within the scope of intelligent transportation systems (ITS) [14]. In a study on automobiles, 3D point cloud-based processing and learning methods were analyzed in autonomous vehicles [15]. White et al. proposed a system to detect traffic accidents and shorten emergency response times, utilizing accelerometer and acoustic sensor data from smartphones to transmit information to a central emergency service, including location information, photographs, and VoIP communication. In this way, they showed that the death rate can be reduced by 56% and that it is a low-cost proposal [16]. Zahid et al. They stated that the most significant deficiency in accident detection is the lack of training data and generated data, which can be simulated on a scene-by-scene basis using artificial intelligence. They trained AlexNet, GoogleNet, SqueezeNet, and ResNet-50 models with this data and showed that AlexNet successfully detected real accidents with an 80% actual positive rate in the UCF-Crime dataset [17]. Wu and Li proposed a CNN architecture to detect accidents and broken windows using a customized dataset. The F1 scores obtained with the proposed architecture showed 62% success for rear window damage, 63% for side window damage, and 83% for windshield damage [18]. Adewopo et al. performed real-time detection by integrating RGB frames and optical flow information to detect accidents, such as rear-end impacts, T-bon crashes, and front impacts, in their proposed model. The detections were made correctly with an average accuracy (mAP) of 87% [19]. Zhou et al. proposed a multilayer neural network architecture that performs time-space-based feature coding for accident detection from in-car cameras. With their method, they first cluster the frames by coding the time information in the videos, and then determine the boundary frames as potential accident moments. Then, the spatial relationships of the objects in these frames are analyzed to detect real accidents. Extensive experiments have demonstrated that this method achieves 79.30% accuracy and efficiency, making it suitable for real-time accident detection in VANET environments [20]. Abbasi et al. proposed a Reversible Data Hiding in Encrypted Images (RDHEI) approach for privacy protection in IoT-based e-health, smart cities, and smart robotics applications. They achieved high embedding capacity and energy efficiency by using multi-MSB based dynamic quadtree partitioning and improved Huffman coding [21]. Shi et al. proposed a novel method combining GAN, XGBoost, and SHAP for the real-time detection of traffic accidents at urban intersections. In the study, synthetic accident data were generated using Generative Adversarial Networks (GAN) to solve the data imbalance problem, and thus, the XGBoost model was trained on a balanced dataset. The model’s performance showed a significant improvement compared to previous methods, achieving a 0.844 AUC with a 7.1% increase. In addition, model outputs were interpreted using Shapley Additive Explanations (SHAP) analysis, which determined that features such as the vehicle-time ratio and average speed were the most critical variables in accident detection [22]. Xu et al. created a comprehensive traffic accident video dataset, called TAD, to address the limitations of existing datasets in intelligent transportation. This dataset comprises authentic accident images captured by surveillance cameras and has been evaluated using standard computer vision algorithms in various tasks, including image classification, video classification, and object detection. In experimental studies, 78% accuracy was achieved in the video classification task using the Swin Transformer model [23]. Dogru and Subası proposed models that will distinguish accidents from everyday situations using Artificial Neural Networks (ANN), Support Vector Machines (SVM) and Random Forest (RF) algorithms to detect accidents and send warnings to drivers by using the speed and location information of the vehicles in the simulated Vehicular Ad-Hoc Networks (VANET) environment. According to the results, the RF algorithm showed the best performance with an accuracy rate of 91.56%; this rate was reported as 90.02% for ANN and 88.71% for SVM [24]. Kamijo et al. developed the time-space MRF method, which addresses the issue of vehicles partially or wholly blocking each other using the Markov random field method. This algorithm can track and segment vehicles with a success rate of 93–96% by modeling the state transitions of pixels both in the image plane and along the time axis. Based on this success, an event recognition system based on the hidden Markov model (HMM) was developed, which detected events such as collisions, transitions, and jams by learning vehicle behavior patterns [25]. Yas et al. developed a yolo-based model to detect vehicles traveling in the opposite direction or on roads. The study compared different models. The researchers observed that YOLOv8 performed better than RCNN [26]. In addition to traffic planning, studies are also carried out on the location and planning of charging and refueling stations [27].

Considering the studies in the literature, traffic accidents are among the most important problems that smart cities need to address. Therefore, automatically detecting accidents that may occur in smart cities and integrated autonomous vehicles is crucial for more effective traffic and health planning. In this paper, a deep learning-based feature extraction and model fusion approach was used using a dataset containing multi-class traffic situations and accidents. Feature maps obtained using transformer and CNN-based models were classified using various machine learning classifier, and the CNN and transformer models with the highest success rates were identified. The proposed model was aimed to achieve more successful results by combining the features extracted from the determined CNN model and the transformer model. The final success rate was then determined using six different classifiers. Consequently, traffic situations were automatically detected by combining the strengths of both CNN and transformer models. The results of the proposed model were compared with those obtained by classifying features from ten different models using six different classifiers. The proposed model was observed to be more successful than the results obtained in the study. In the second part of this study, detailed information about the dataset used, the general structure of the proposed model, and the architectures employed are provided. In Section 3, experimental results and comparisons obtained by using the proposed architecture with the dataset are included. In the Discussion section, the evaluation of the model’s success and its future perspective is explained. Finally, the conclusion section is presented.

## 2. Materials and methods

### 2.1 Dataset

The Traffic-Net dataset comprises a total of 4,400 labeled images, prepared for visual recognition and classification tasks related to traffic. The dataset is divided into four primary classes: sparse traffic, heavy traffic, accidents, and fires. The images are collected from various online platforms, including Google Images, Bing, and Flickr, and have different resolutions and sizes. Each class contains examples that represent various situations, ranging from traffic signs to emergency events. The number of data points in each class is balanced at 1100. Since the number of data is sufficient, data augmentation was not performed in the study. Traffic-Net is a comprehensive dataset designed for computer vision applications, including image classification, object detection, and segmentation [28].

### 2.2 Proposed model

**Fig 1:**
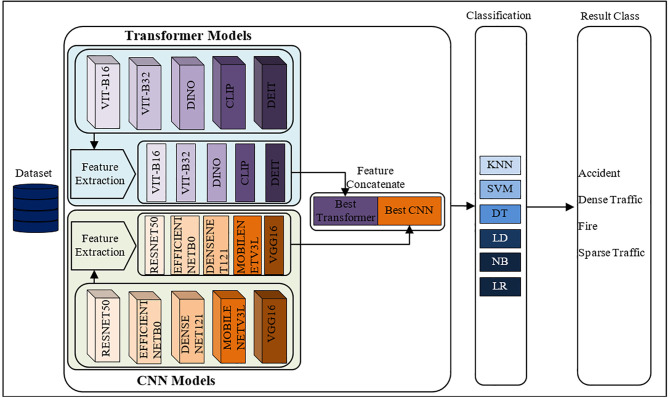
Proposed model for traffic status detection.

Automatic detection of traffic events is essential for the sustainability of smart cities and the improvement of quality of life. Artificial intelligence-supported decision support systems exhibit strong performance, especially in extracting meaningful information from visual data. In this context, the primary purpose of the model developed in this study is to contribute to the safety, efficiency, and sustainability goals in smart city infrastructures by providing accurate automatic detection of traffic events. The proposed model is presented in Fig 1.

In the proposed model, feature extraction is performed using a total of ten deep learning architectures. These architectures consist of five CNN models and five Transformer-based models. In the first stage, feature extraction is performed separately from each CNN architecture, and the best-performing CNN architecture is determined by using these features. Similarly, the features obtained from transformer architectures are classified among themselves, and the best transformer architecture is determined. The features of the most effective CNN and transformer architectures are merged in the second stage, and the resulting feature set is utilized for the final classification procedure. By combining the contextual relationship-capturing ability of transformer models with the spatial learning capabilities of CNN models, this technique aims to enhance classification accuracy.

### 2.3 Convolutional Neural Network (CNN)

CNN architectures are a deep learning based architecture that is frequently used especially in the field of image classification. Convolution layers are one of the most basic layers of CNN architectures. In particular, feature extraction is performed in this layer. CNNs can achieve serious success in subjects such as object detection, segmentation and classification. In this study, CNN architectures were used as a basis to extract features from images in the traffic dataset. The classification abilities of the architectures on the data were compared using several CNN models with various structural characteristics. In this context, ResNet50 [29], EfficientNetB0 [30], DenseNet121 [31], MobileNetV3L [32] and VGG16 [29] CNN architectures were used. The features extracted from these models were evaluated for classification in the later stages, and the best-performing CNN architecture was determined.

### 2.4 Transformers

The Transformer architecture has also demonstrated remarkable effectiveness in image processing, building upon research in the field of natural language processing. When employing transformer topologies, information beyond the most significant features in the data is removed to facilitate the study. Specifically, the attention mechanism and Multi-Head Self-Attention modules make up the fundamental transformer structure [33]. In artificial neural networks, the attention mechanism, which was inspired by the human ability to focus on important information in daily life, functions similarly to ignore unimportant information. While Bahdanau attention is used in language translation, it aims to highlight essential features by removing unnecessary features in the image with methods such as Compression and Stimulation in image processing. The self-attention mechanism operates by considering the relationship between the query, key, and value vectors, which are calculated in parallel at the matrix level of the model input. The multi-head attention structure tries to learn different features by applying more than one self-attention head in parallel to the same input data in transformers [33]. This structure creates a multi-layered system with various relationships and a representation that emerges from their combination.

#### 2.4.1 Transformers architectures.

**Fig 2:**
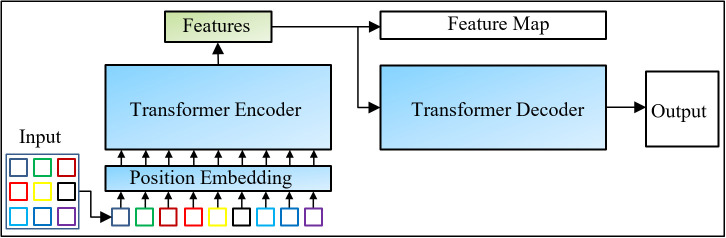
Transformer general structure and feature extraction stage.

Transformers consist of two basic structures: encoder and decoder. The encoder produces a vector representing each input data, while the decoder produces output using these vectors. The encoder structure of a general transformer includes a multi-head attention mechanism, a feedforward network structure, and normalization steps with residual connections in each layer. The decoder structure, unlike the encoder structure, features an additional self-attention layer that enables the production of new output by considering past outputs. At the end of the decoder, the output is produced using a linear layer and a Softmax activation function. The structure of a basic transformer model is given in Fig 2.

#### 2.4.2 Visual transformer models.

As an alternative to CNN architectures in image classification, segmentation, and object detection, ViT architectures are a key starting point for developments in this field. ViT architectures divide images into small pieces to learn spatial information from the image and provide it as input to the transformer architecture. By modifying the configurations of ViT architectures, different variants were employed, including ViT-Base-Patch16–224 [34], which works with 16x16 patches, and ViT-Base-Patch32–224 [35], which works with 32x32 patches, for feature extraction in this study. A data-efficient Image Transformer DeiT model was developed to address the limitations of ViT models, which require excessive data in cases with limited data availability. The DeiT-Base-Patch16–224 [36] model was used for feature extraction in this study. The DINOv2 [37] model is another architecture used for feature extraction in this study, as it can extract meaningful features without the need for labeled data, thanks to its self-supervised structure. The CLIP model is an architecture that can learn to combine text and images. Considering this feature, feature extraction was performed using the CLIP-ViT-B/32 [38] model.

### 2.5 Classifiers

The results were obtained to analyze the classification performance of the architectures whose features were extracted. Six different classifiers were used at this stage. These classifiers are the K-Nearest Neighbors (KNN) algorithm [39], which classifies the samples according to their nearest neighbors without requiring a training process, the Cubic Kernel Support Vector Machine (Cubic SVM) [40], which classifies the data by determining the best separating line or hyperplane, the Decision Tree (DT) [41], which divides the data into branches according to decision rules, the Linear Discriminant Analysis (LDA) [42], which establishes a linear relationship between the samples and the class labels, and the Naive Bayes (NB) [43] algorithm, which assumes that each feature is independent from each other and performs the classification based on probability. The performances of these classifiers were analyzed using different metrics. Each of these performance metrics is used to evaluate the model’s performance from different aspects. Thus, it is possible to make accurate analyses of the model performance. The accuracy metric determines the proportion of correct predictions of the model among all predictions. Precision indicates that the data predicted as positive in the prediction results are indeed positive. False positive rate is used in cases where there is a significant imbalance. Recall is used to determine how many of the true positive examples were predicted correctly. The recall value must be high to prevent false negatives from producing biased results. Finally, the F1-score is calculated by taking the harmonic mean of the precision and recall metrics. The F1-score is a preferred metric in imbalanced data sets. The performance of the models is measured using performance evaluation metrics.

## 3. Experimental results

The findings of the experimental investigations conducted as part of the proposed approach for categorizing 4-class data in the TrafficNet dataset are presented in this section. Ten deep learning models were used in the study, including five CNN architectures and five Transformer-based architectures, to extract significant characteristics from image data. The experiment planning consisted of two phases. In the first step, the features of each CNN and Transformer model were extracted independently, and performance assessments were conducted using different classification methods with these features. The CNN and Transformer models with the best success rates were then identified. After combining the features from these two top models, the final classification procedure was performed on the combined feature set. This strategy aims to increase classification success by combining the strong spatial representation capabilities of CNN models with the ability of Transformer architectures to learn contextual relationships.

All model execution, feature extraction and all steps of the proposed model were calculated using NVIDIA Tesla T4 GPU on the Google Colab platform. For all CNN and Transformer architectures, 3600 training and 800 test samples were used, and the feature size varied according to the architecture. While 768-dimensional features were extracted for each of the transformer-based models ViT, DINO, DEIT, 512-dimensional features were extracted for the CLIP model. For CNN-based architectures ResNet50, EfficientNetB0, DenseNet121, MobileNetV3-Large and VGG16, 1000-dimensional features were extracted from each architecture. In order to improve the performance of the classifications, CLIP, which shows the best performance among Transformer-based architectures, and MobileNetV3-Large, which shows the best performance among CNN-based architectures, were combined. As a result of combining the features of these two models, a total of 1512-dimensional combined feature vector was obtained for each sample. Below, the results obtained by each model, along with the comparative analyses, are presented in detail.

### 3.1 ResNet50 – Classifiers results

The first model used in the study to compare the performance of the proposed model is the ResNet50 model. The features extracted from this model, along with the performance of different classifiers on the test data, are shown in Table 1.

**Table 1:** Performance of feature maps obtained with ResNet50 in classifiers.

ResNet50	ACC % (Test)	Error Rate % (Test)	Weighted Precision % (Test)	Weighted Recall % (Test)	Weighted F1 Score % (Test)	Prediction Speed (obs/sec)	Training Time (sec)
KNN	87.25	12.75	87.47	87.25	87.32	3728.93	0.02
SVM	91.62	8.38	91.78	91.62	91.65	1945.62	1.82
DT	74.25	25.75	74.04	74.25	74.08	797585.74	7.20
LD	91.12	8.88	91.44	91.12	91.21	72236.19	0.94
NB	83.62	16.38	84.00	83.62	83.56	73542.35	0.03

Table 1 shows that SVM produced the highest accuracy rate (91.62%) and F1 score (91.65%). The LD classifier, which produces comparable performance numbers, comes next. With an accuracy of 74.25%, the DT classifier had the lowest success rate. Fast training time and high prediction speed per observation are two further notable features of the KNN classifier. These findings demonstrate that SVM utilizes ResNet50-based features to achieve the most balanced and efficient classification performance.

### 3.2 EfficientNetB0 – Classifiers results

The second CNN-based model used for traffic status detection is EfficientNetB0. The EfficientNetB0 architecture, developed by Google, stands out with its compound scaling method, which optimizes the scaling process. This approach enhances the depth, width, and input resolution of the model in a balanced manner, enabling higher accuracy to be achieved with fewer parameters. The results obtained in this model are in Table 2.

**Table 2:** Performance of feature maps obtained with EfficientNetB0.

Efficient NetB0	ACC % (Test)	Error Rate % (Test)	Weighted Precision % (Test)	Weighted Recall % (Test)	Weighted F1 Score % (Test)	Prediction Speed (obs/sec)	Training Time (sec)
KNN	88.75	11.25	88.87	88.75	88.73	4240.64	0.01
SVM	91.00	9.00	91.30	91.00	91.04	1626.20	2.04
DT	75.38	24.62	75.58	75.38	75.46	975703.17	7.07
LD	92.88	7.12	92.92	92.88	92.89	420376.25	0.84
NB	84.75	15.25	85.32	84.75	84.77	66211.04	0.02

Table 2 presents the comparative classification performances obtained with the features extracted from the EfficientNetB0 model. The highest accuracy rate of 92.88% and F1 score of 92.89% were obtained in the LD classifier. SVM followed LD with an accuracy rate of 91.00% and an F1 score of 91.04%. The lowest success rate was observed in the DT classifier with an accuracy rate of 75.38%. While KNN stands out as the classifier with the fastest training time, the LD classifier stands out with both its high success rate and balanced metric values. These results indicate that the EfficientNetB0 model is most effectively utilized with the LD classifier.

### 3.3 DenseNet121 – Classifiers results

DenseNet121 is a model based on the dense connection principle, where each layer is directly connected to all previous layers. This model improves gradient propagation while increasing parameter efficiency. When the features obtained with this model used for traffic flow were classified with different classifiers, the metrics in Table 3 were obtained.

**Table 3:** Performance of feature maps obtained with DenseNet121.

DenseNet121	ACC % (Test)	Error Rate % (Test)	Weighted Precision % (Test)	Weighted Recall % (Test)	Weighted F1 Score % (Test)	Prediction Speed (obs/sec)	Training Time (sec)
KNN	86.25	13.75	86.43	86.25	86.31	4517.65	0.02
SVM	90.75	9.25	90.95	90.75	90.77	2116.74	1.53
DT	73.50	26.50	73.37	73.50	73.42	984867.39	7.40
LD	91.88	8.12	92.12	91.88	91.93	57442.45	0.82
NB	84.38	15.62	84.65	84.38	84.41	66733.82	0.03

Table 3 presents the comparative test performances of different classifiers with the features extracted from the DenseNet121 model. The highest accuracy rate was achieved with the LD classifier, at 91.88%, and an F1 score of 91.93%. SVM achieves 90.75% accuracy and a 90.77% F1 score, following LD. The lowest performance was observed in the DT classifier, with an accuracy of 73.50%. While KNN stands out with its fast training time and high prediction speed, the LD classifier, DenseNet121, presented the most effective results in terms of overall success, given its features.

### 3.4 MobileNetV3L – Classifiers results

MobileNetV3-Large is a lightweight and efficient model optimized to run with high accuracy on mobile and embedded devices. When the features obtained with this model used for traffic flow were classified with different classifiers, the metrics in Table 4 were obtained.

**Table 4:** Performance of feature maps obtained with MobileNetV3L.

MobileNet V3L	ACC % (Test)	Error Rate % (Test)	Weighted Precision % (Test)	Weighted Recall % (Test)	Weighted F1 Score % (Test)	Prediction Speed (obs/sec)	Training Time (sec)
KNN	86.50	13.50	86.58	86.50	86.52	4118.78	0.01
SVM	93.00	7.00	93.09	93.00	93.02	2765.85	1.23
DT	73.38	26.62	73.46	73.38	73.40	958150.54	7.23
LD	92.12	7.88	92.28	92.12	92.16	420428.92	0.83
NB	76.75	23.25	77.11	76.75	76.52	75530.52	0.03

Table 4 shows the classification performances obtained using the features extracted from the MobileNetV3-Large model, compared to other models. The highest accuracy rate was achieved with 93.00%, and the F1 score was 93.02% in the SVM classifier. LD follows SVM with 92.12% accuracy and 92.16% F1 score. The lowest performance was observed in the DT classifier, with an accuracy of 73.38%. The results of the NB classifier were also at a low level, whereas KNN again attracted attention with its fast training time. In general, the SVM classifier gave the most accuracy rate with the features obtained from the MobileNetV3L model.

### 3.5 VGG16 – Classifiers results

VGG16 is an architecture that increases depth by using small fixed-size filters and offers high accuracy despite its simple structure. VGG16 is widely preferred in image classification and transfer learning tasks. When the features obtained with this model used for traffic flow were classified with different classifiers, the metrics in Table 5 were obtained.

**Table 5:** Performance of feature maps obtained with VGG16.

VGG16	ACC % (Test)	Error Rate % (Test)	Weighted Precision % (Test)	Weighted Recall % (Test)	Weighted F1 Score % (Test)	Prediction Speed (obs/sec)	Training Time (sec)
KNN	82.50	17.50	82.77	82.50	82.42	4815.39	0.01
SVM	89.12	10.88	89.27	89.12	89.16	2113.47	1.59
DT	75.00	25.00	75.17	75.00	75.06	682972.36	7.27
LD	89.25	10.75	89.45	89.25	89.30	57405.11	2.32
NB	81.62	18.38	81.86	81.62	81.62	76655.55	0.03

**Fig 3:**
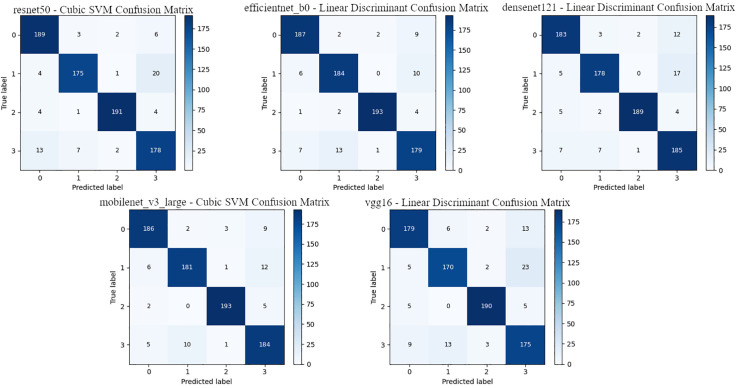
CNN architectures – confusion matrix of the best classifier.

Table 5 presents the classification results obtained using the features extracted from the VGG16 model, compared to other models. The highest accuracy rate was achieved with the LD classifier, at 89.25%, and an F1 score of 89.30%. LD is followed by the SVM classifier, yielding very close values of 89.12% accuracy and 89.16% F1 score. The lowest performance was observed in the DT classifier, with an accuracy of 75.00%. While KNN stands out with its fast training time and prediction speed, the LD classifier provided the most efficient result with the VGG16 features in terms of overall success. The confusion matrices for the classifiers, where CNN architectures are the most successful, are shown in Fig 3.

When Fig 3 is examined, DenseNet121 and EfficientNetB0 produced quite balanced and successful results with the Linear Discriminant classifier, and classified the images in class 2 almost without error. MobileNetV3 provided similarly high accuracy with SVM, and stood out with 193 correct classifications for class 2. Although ResNet50 was successful with the Cubic SVM classifier, it made relatively more errors for class 3. VGG16, on the other hand, showed more classification errors than the other models, and especially had higher error rates in classes 1 and 3.

### 3.6 VIT-B16 – Classifiers results

The ViT-B16 model is one of the first models to utilize the Transformer architecture, which was initially developed for image classification. In this model, the input image is divided into 16 × 16 pixel patches, and each patch is converted into a series of vectors, which are then fed to Transformer blocks. Using this model, the feature maps obtained from the traffic dataset are classified using different classifiers, and the results are presented in Table 6.

**Table 6:** Performance of feature maps obtained with VIT-B16.

VIT-B16	ACC % (Test)	Error Rate % (Test)	Weighted Precision % (Test)	Weighted Recall % (Test)	Weighted F1 Score % (Test)	Prediction Speed (obs/sec)	Training Time (sec)
KNN	87.88	12.12	88.35	87.88	87.87	4730.97	0.01
SVM	94.25	5.75	94.41	94.25	94.28	2819.73	1.20
DT	75.62	24.38	75.64	75.62	75.62	670016.61	5.41
LD	92.62	7.38	92.86	92.62	92.69	316431.84	1.66
NB	84.62	15.38	84.90	84.62	84.58	79709.31	0.02

Table 6 presents the classification performances obtained using the features extracted from the VIT-B16 model, compared to other models. The highest accuracy rate was achieved at 94.25%, and the F1 score was 94.28% in the SVM classifier. LD follows SVM with 92.62% accuracy and 92.69% F1 score. The lowest success rate was observed in the DT classifier, with an accuracy rate of 75.62%. While KNN stands out with its high prediction speed and low training time, the performance of the NB classifier remained at a moderate level. The general results show that the VIT-B16 model achieved the highest success with the SVM classifier.

### 3.7 VIT-B32 – Classifiers results

The ViT-B32 model, a transformer-based visual classification model, is a different version of the ViT-B16 model. This model processes the input images by dividing them into larger patches of 32 × 32 pixels. In this way, the processing cost of the model decreases, while the loss of detailed spatial information increases slightly. When the features obtained with this model used for traffic flow were classified with different classifiers, the metrics in Table 7 were obtained.

**Table 7:** Performance of feature maps obtained with VIT-B32.

VIT-B32	ACC % (Test)	Error Rate % (Test)	Weighted Precision % (Test)	Weighted Recall % (Test)	Weighted F1 Score % (Test)	Prediction Speed (obs/sec)	Training Time (sec)
KNN	89.50	10.50	89.65	89.50	89.53	5173.84	0.01
SVM	93.88	6.12	94.02	93.88	93.89	2859.26	1.42
DT	76.50	23.50	76.66	76.50	76.56	902728.87	4.48
LD	93.12	6.88	93.21	93.12	93.14	277332.28	0.46
NB	84.12	15.88	84.93	84.12	84.18	97809.22	0.02

Table 7 presents the performance of different classifiers using features extracted from the VIT-B32 model. The highest accuracy rate was achieved with the SVM classifier, at 93.88%, and an F1 score of 93.89%. This was followed by the LD classifier, which achieved 93.12% accuracy and a 93.14% F1 score. The lowest success was observed in the DT classifier with 76.50% accuracy. While KNN stands out as the classifier with the fastest prediction time, the NB classifier showed a moderate performance. In general, it is observed that the VIT-B32 model achieves the highest classification success in conjunction with the SVM classifier.

### 3.8 DINO – Classifiers results

DINO is a method that aims to learn strong visual representations without using labels. However, models pre-trained with DINO can be used directly or fine-tuned in downstream tasks with labeled data. The performance metrics of this model used for feature extraction from the dataset in this study are presented in Table 8.

**Table 8:** Performance of feature maps obtained with DINO.

DINO	ACC % (Test)	Error Rate % (Test)	Weighted Precision % (Test)	Weighted Recall % (Test)	Weighted F1 Score % (Test)	Prediction Speed (obs/sec)	Training Time (sec)
KNN	93.38	6.62	93.37	93.38	93.37	5832.57	0.01
SVM	94.62	5.38	95.05	94.62	94.67	3518.15	0.98
DT	82.62	17.38	82.63	82.62	82.61	699342.06	5.00
LD	95.00	5.00	95.17	95.00	95.02	57451.30	0.73
NB	90.50	9.50	91.43	90.50	90.69	79512.87	0.02

Table 8 presents the results of the classification operations performed using the features extracted from the DINO model, comparatively. The highest accuracy rate was obtained at 95.00% with an F1 score of 95.02% in the LD classifier. LD is followed by SVM, with very close performance, achieving 94.62% accuracy and 94.67% F1 score. The lowest success rate was observed in the DT classifier, with an accuracy of 82.62%. While the KNN classifier attracted attention with its high accuracy of 93.38% and speedy prediction time, the NB classifier also showed a satisfactory performance. In general, the highest classification success was achieved with the features obtained from the DINO model using LD.

### 3.9 CLIP – Classifiers results

CLIP is a multimodal model that maps images and text into a common representation. Visual and linguistic encoders are trained together to place image-text pairs with the same meaning into close vectors and different ones into far vectors. With this structure, CLIP can classify and explain any image in response to natural language queries. The performance metrics of this model used for feature extraction from the dataset in this study are presented in Table 9.

**Table 9:** Performance of feature maps obtained with CLIP.

CLIP	ACC % (Test)	Error Rate % (Test)	Weighted Precision % (Test)	Weighted Recall % (Test)	Weighted F1 Score % (Test)	Prediction Speed (obs/sec)	Training Time (sec)
KNN	95.00	5.00	95.02	95.00	94.99	7958.23	0.00
SVM	96.62	3.38	96.76	96.62	96.63	7057.92	0.45
DT	86.50	13.50	86.75	86.50	86.49	1132830.25	2.84
LD	96.50	3.50	96.54	96.50	96.50	744000.71	0.22
NB	93.50	6.50	94.33	93.50	93.57	94824.03	0.01

Table 9 presents the performance of different classifiers with features extracted from the CLIP model. The SVM classifier achieved the highest accuracy rate of 96.62% and an F1 score of 96.63%. SVM was followed by LD, yielding very close results with 96.50% accuracy and a 96.50% F1 score. The lowest performance was observed in the DT classifier, with an accuracy of 86.50%. KNN gave very successful results with 95.00% accuracy and was the classifier with the fastest prediction time. The NB classifier also demonstrated good overall performance, achieving a 93.57% F1 score. These results show that the CLIP model achieved the highest classification success in conjunction with the SVM classifier.

### 3.10 DEIT – Classifiers results

DeiT is a data-efficient image classification model developed based on the Vision Transformer architecture. Unlike other ViT models, it achieves high accuracy by training only with medium-sized datasets, such as ImageNet, and exhibits comparable performance to CNN-based models. In addition, DeiT is supported by knowledge distillation (training model transfer) and provides better learning with limited data by utilizing the training CNN model during the training process. The performance metrics of this model used for feature extraction from the dataset in this study are presented in Table 10.

**Table 10:** Performance of feature maps obtained with DEIT.

DEIT	ACC % (Test)	Error Rate % (Test)	Weighted Precision % (Test)	Weighted Recall % (Test)	Weighted F1 Score % (Test)	Prediction Speed (obs/sec)	Training Time (sec)
KNN	44.00	56.00	44.25	44.00	43.87	6901.42	0.01
SVM	56.50	43.50	57.25	56.50	56.56	1154.87	3.34
DT	43.25	56.75	43.61	43.25	43.34	894307.89	5.01
LD	58.75	41.25	58.51	58.75	58.58	61832.11	0.47
NB	37.75	62.25	36.14	37.75	32.66	83741.63	0.02

**Fig 4:**
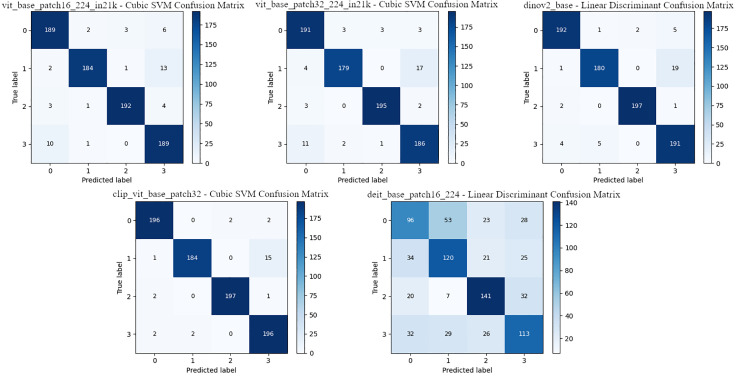
ViT architectures – confusion matrix of the best classifier.

Table 10 presents the comparative classification results, which include the features extracted from the DEIT model. The highest accuracy rate was achieved with the LD classifier, at 58.75%, and an F1 score of 58.58%. SVM followed LD with 56.50% accuracy and 56.56% F1 score. The lowest performance was observed in the NB classifier with 37.75% accuracy and 32.66% F1 score. KNN and DT classifiers also showed low success. These results indicate that the features extracted from the DEIT model are not sufficiently discriminative for classification and reveal that the classification performance of DEIT is significantly lower compared to other transformer models. The confusion matrices for the classifiers, where ViT architectures are the most successful, are shown in Fig 4.

The confusion matrices presented in Fig 4 show the results obtained by the best classifiers using features extracted from the dataset via ViT architectures. In general, the ViT-B16, ViT-B32, DINOv2, and CLIP-ViT models demonstrated high classification performance across all four classes. Especially CLIP-ViT-B32 stands out by providing almost error-free classification in classes 0 and 3. The DeiT-B16 model showed significantly lower success compared to the other models.

### 3.11 Results of the proposed model

A hybrid model was developed for detecting traffic conditions. The developed hybrid model was selected among 5 CNN and 5 ViT architectures accepted in the literature. First, feature maps of the images in the dataset were extracted with 10 different models. The extracted features were classified into five different classifiers. The CNN and ViT architectures that achieved the highest performance were used as the base in the proposed model. Clip and MobileNetV3 architectures achieved the highest performance. Then, the feature maps obtained from these architectures were combined to bring together different features of the same image. These CNN and ViT-based features were classified using different classifiers. The highest performance was obtained in the LD classifier. The performance metrics of the proposed model are in Table 11.

**Table 11:** Performance metrics of the proposed model for traffic status detection.

Proposed Model	ACC % (Test)	Error Rate % (Test)	Weighted Precision % (Test)	Weighted Recall % (Test)	Weighted F1 Score % (Test)	Prediction Speed (obs/sec)	Training Time (sec)
KNN	87.00	13.00	87.06	87.00	87.02	3137.49	0.02
SVM	94.38	5.62	94.47	94.38	94.38	1677.74	11.31
DT	82.50	17.50	82.73	82.50	82.57	871996.67	9.11
LD	**96.88**	3.12	96.90	96.88	96.87	162696.04	1.85
NB	92.00	8.00	92.45	92.00	92.03	47301.07	0.03

**Fig 5:**
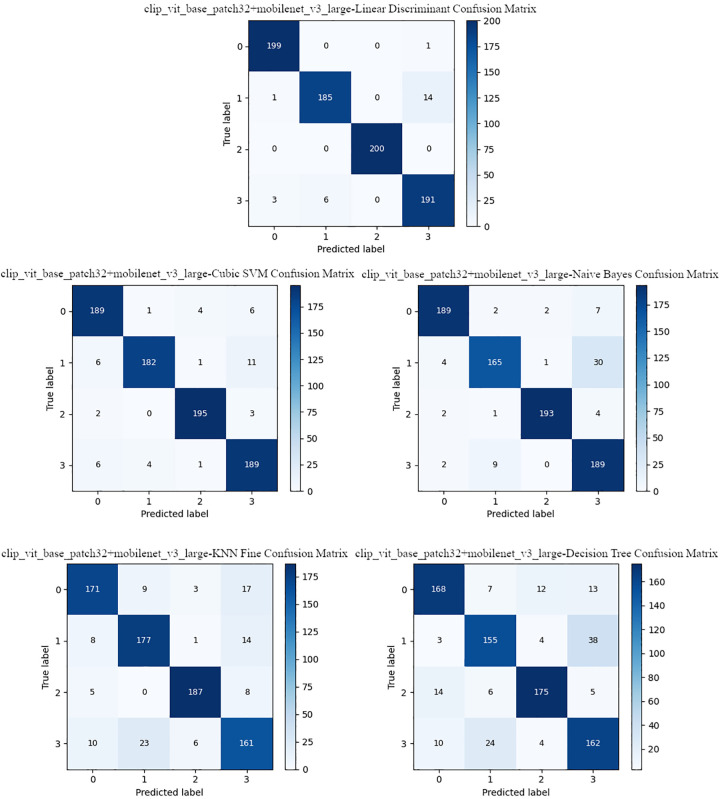
Classifier confusion matrices for the proposed model.

Table 11 presents the classification performances obtained by combining the features extracted from CLIP and MobileNetV3-Large models within the scope of the proposed model. The highest accuracy rate of 96.88% and the F1 score of 96.87% were obtained in the LD classifier. SVM achieves 94.38% accuracy and a 94.38% F1 score, following LD. The lowest accuracy is observed in the DT classifier with 82.50%. While KNN stands out with its fast prediction time, the NB classifier offered a strong alternative with 92.00% accuracy. These results demonstrate that the classification performance is significantly improved when the contextual learning power of CLIP is combined with the efficient spatial feature extraction capabilities of MobileNetV3. Confusion matrices of the proposed model for different classifiers are shown in Fig 5.

The most accuracy rate were obtained with the proposed model in the LD classifier, while the least successful results were achieved in the DT classifier. The proposed model achieved high accuracy rates for four classes in the LD classifier. In general, the model exhibits intense inter-class discrimination, and the results indicate that multi-model-based feature fusion is effective in enhancing classification performance.

## 4. Discussion

Automatic detection of traffic conditions is of critical importance, as it is a fundamental component of modern smart city applications. Traffic monitoring and evaluation processes carried out using traditional methods have disadvantages, including high labor requirements, time loss, and limited scalability. In contrast, automatic systems based on artificial intelligence and image processing techniques can quickly and accurately detect events such as traffic density, accidents, and fires by performing real-time analysis. In this case, transportation safety is increased and intervention times are shortened. In addition, when these systems are supported by big data analytics, traffic flow optimization, carbon emission reduction, and more efficient use of transportation resources become possible. Therefore, automatic traffic condition detection stands out as an indispensable component of creating a sustainable, safe, and efficient transportation infrastructure. In this study, a hybrid model has been developed for detecting traffic conditions. The current ViT and CNN architectures are combined in the developed model. The feature maps obtained with these architectures are classified using different classifiers by combining various features of the same image. The proposed model is compared with current studies in Table 12 and also with five ViT and five CNN architectures. The obtained results show that the proposed model achieves successful results in traffic status detection.

**Table 12:** Literature review.

Author(s)	Method/ Model	Data Type	Contribution/ Feature	Success/ Performance
Ali et al. [5]	OLDA + Bi-LSTM + FastText	Social media data	Ontology-based event recognition, case detection with sentiment analysis	%97 accuracy
Chen et al. [6]	TCFF-GAN	Video (SO-TAD dataset)	Square estimation with cosine similarity	71.25% AUC
Mariano et al. [7]	XGBoost	Radar sensör verisi	TTDA reduction with neighboring sensors	%83 AUC, %49 precision
Kumar et al. [9]	HOG + DML	Road segmentation data	DML supported model using AADT	%95.4 accuracy
Nusari et al. [10]	YOLOv9-C, YOLO-NAS-L	Real time video	Real-time CNN based detection	%92.7 (YOLOv9-C), %85 (YOLO-NAS-L) mAP
Yu et al. [13]	Video 3D Transformer	Dashcam video	Fine-level analysis (genre, time-space, intensity)	Successful in different tasks
Vinodhini et al. [14]	LoRa + sensor + GPS	IoT sensor data	Low latency, human intervention-free system	Time saving, low latency
White et al. [16]	Smartphone sensors	Mobil sensor data	Instant notification and scene awareness	False positive reduction
Zahid et al. [17]	AlexNet	Simule + real-time video	Training on real accidents with simulated data	%80 TPR (AlexNet)
Wu and Li [18]	CNN	Video	Classification according to glass damage types	83% (windshield), 63% (side window) F1
Adewopo et al. [19]	I3D-CONVLSTM2D	Video	Lightweight model, smart city compatibility	%87 mAP
Zhou et al. [20]	Spatio-temporal MLP	VANET	Square clustering with occlusion solution	Real time, high accuracy
Shi et al. [22]	GAN + XGBoost + SHAP	Intersection camera data	Data imbalance resolution, variable analysis	AUC %0.844
Xu et al. [23]	Swin Transformer	TAD dataset (video)	Open dataset focused on highways	%78 accuracy
Doğru and Subaşı [24]	RF, ANN, SVM	Simulated speed/position data	Detection by data exchange between vehicles	RF: %91.56, ANN: %90.02, SVM: %88.71
Kamijo et al. [25]	Spatio-temporal MRF + HMM	Intersection camera view	Occlusion resolution and event recognition	%93–96 follow-up success
Proposed Model	Clip+ ModileNetV3	Camera View	Classification with Transformer and CNN integration	%96.88 accuracy

Table 12 presents a comparative performance analysis of the proposed model with various methods from the literature. When compared with studies conducted with various data types and models, the proposed model has achieved a high level of success with an accuracy rate of 96.88%. The effective use of both contextual and spatial information, thanks to the combination of Transformer and CNN-based architectures, makes the model a strong candidate, especially in image-based classification tasks. The comparison with many studies in the literature reveals that the proposed model offers a competitive and innovative approach. However, it has some limitations. The proposed model has been evaluated only on the Traffic-Net dataset. This limitation may lead to potential biases due to the dataset’s inability to comprehensively capture the variability present in real-world traffic scenarios. Factors such as changes in lighting conditions, weather conditions, camera perspectives, and scene complexity can significantly impact model performance when used in practical applications. To improve the model’s performance and generalizability, our goal in future work is to evaluate the model on multiple publicly available traffic datasets encompassing various environmental conditions and viewpoints. We also plan to validate the model under real-world conditions using video data collected from different urban environments. Furthermore, due to the time-sensitive nature of traffic monitoring, future work will focus on optimizing inference speed and computational efficiency to ensure the model’s suitability for real-time deployment in smart city environments.

## 5. Conclusion

Automatic detection of traffic conditions is essential for efficient management of urban transportation, rapid response to emergencies, and reduction of environmental impacts. In this study, multiple features obtained from CLIP-ViT-B32 and MobileNetV3-Large models, which perform deep learning-based visual feature extraction, were combined. This combination successfully separated four classes related to traffic conditions using an LD classifier. The exceptionally high accuracy rates and minimal confusion between classes demonstrate that the proposed method possesses a strong discriminative ability. These findings are valuable not only from an academic perspective but also from urban planning, traffic engineering, and public safety perspectives. Automatic traffic detection has the potential to be applied in various areas, including traffic light optimization, accident management, emergency response planning, and environmentally friendly transportation strategies. In the future, training such systems with larger and more heterogeneous datasets will enable the development of more general, flexible, and scalable solutions that take into account variables such as different weather conditions, time zones, and geographical regions. In addition, integrating such systems with real-time data flow and testing them in the field will accelerate the transition from prototype to application, thereby accelerating the adoption of smart city technologies.
